# Supplementary material for: Toward secure mobile applications through proper authentication mechanisms
Source: PLoS One. 2024 Dec 5;19(12):e0315201. doi: 10.1371/journal.pone.0315201 (PMC11620593; doi:10.1371/journal.pone.0315201)
Supplement: Attachment — Submitted filename: renamed_5fe45.docx [file pone.0315201.s001.docx]

**Review Comments for ‘Toward Secure Mobile Applications Through Proper Authentication Mechanisms’**

The work is highly relevant to the current landscape, where the rapid rise of mobile apps has brought their integrity, confidentiality, and overall security into sharp focus. With mobile applications transitioning from a luxury to an essential part of daily life, concerns around the protection of sensitive user data are more pressing than ever. As apps increasingly handle personal and financial information, the need for robust security mechanisms is undeniable.

The suggestion of a unified and user-friendly authentication procedure, aimed at reducing the cognitive burden of managing multiple passwords, is a logical approach. Simplifying the login process would enhance user experience and lower the barriers to app adoption. However, this introduces a critical debate: while centralized systems for password management might streamline access, they inherently create single points of failure. In contrast, distributed authority models are seen as more secure, offering resilience by decentralizing control. How does the author debate on this point is the main concern? The paper would be complete if this point is touched upon and debated. These models mitigate the risks of mass data breaches but may come with their own challenges, such as increased complexity in managing security protocols across multiple nodes.

Additionally, the ever-evolving threat landscape, with cyber-attacks growing more sophisticated, makes it imperative to carefully evaluate the strengths and limitations of each approach. It is essential to balance ease of use with the highest level of security. This needs to be justified.

All in all, this paper presents a well-researched, thorough survey of the authentication methods employed by mobile apps today, shedding light on potential future directions and improvements in mobile app security. The exploration of the trade-offs between usability and security, as well as centralized versus distributed control, adds significant value to ongoing discussions in the field.
