# Supplementary material for: Toward secure mobile applications through proper authentication mechanisms
Source: PLoS One. 2024 Dec 5;19(12):e0315201. doi: 10.1371/journal.pone.0315201 (PMC11620593; doi:10.1371/journal.pone.0315201)
Supplement: Attachment — Submitted filename: Response to reviewers.docx [file pone.0315201.s002.docx]

**Response to Reviewers’ Comments**

**Reviewer #1:**

1- **Include a section on research directions in the paper.**

**Answer:** Thanks for this comment. We created a new section (Future Research Directions). We moved the last paragraph of the conclusion section and added a new paragraph to this new section.

2- **Add a reference to the paper 'CMAF-IIoT: Chaotic Map-based Authentication Framework for Industrial Internet of Things' in the text.**

**Answer:** Thanks for this comment. It is an interesting research paper which is related to our work. We added it and talked about it in the last section of Related Work (A).

3- **Insert an abbreviation table in the revised manuscript.**

**Answer:** Thanks for your comment. We agree that we mentioned a lot of abbreviations and thus it might be better to move their detentions to a table. You can find the added table before the references section.

4- **include a few lines discussing the significance of the paper**

**Answer:** Thanks for this comment. We added more information about the significance of our paper before the last paragraph of the introduction.

**Reviewer #2:**

1- **The work is highly relevant to the current landscape, where the rapid rise of mobile apps has brought their integrity, confidentiality, and overall security into sharp focus. With mobile applications transitioning from a luxury to an essential part of daily life, concerns around the protection of sensitive user data are more pressing than ever. As apps increasingly handle personal and financial information, the need for robust security mechanisms is undeniable.**

**Answer:** Thanks for this comment. We are glad that an expert highlights the importance of our study.

2- **The suggestion of a unified and user-friendly authentication procedure, aimed at reducing the cognitive burden of managing multiple passwords, is a logical approach. Simplifying the login process would enhance user experience and lower the barriers to app adoption. However, this introduces a critical debate: while centralized systems for password management might streamline access, they inherently create single points of failure. In contrast, distributed authority models are seen as more secure, offering resilience by decentralizing control. How does the author debate on this point is the main concern? The paper would be complete if this point is touched upon and debated. These models mitigate the risks of mass data breaches but may come with their own challenges, such as increased complexity in managing security protocols across multiple nodes.**

**Answer:** Thanks for this comment. It adds value to our article because it is related to one solution that minimizes cognitive load (i.e. unified authentication). We added a paragraph to show this debate at the end of the discussion section.

3- **Additionally, the ever-evolving threat landscape, with cyber-attacks growing more sophisticated, makes it imperative to carefully evaluate the strengths and limitations of each approach. It is essential to balance ease of use with the highest level of security. This needs to be justified.**

**Answer:** Thanks for this comment. We added one paragraph to address this issue. The paragraph starts with “As e-commerce…” and is located at the end of the discussion section.

4- **All in all, this paper presents a well-researched, thorough survey of the authentication methods employed by mobile apps today, shedding light on potential future directions and improvements in mobile app security. The exploration of the trade-offs between usability and security, as well as centralized versus distributed control, adds significant value to ongoing discussions in the field.**

**Answer:** Thanks for this comment. We added a paragraph about this at the end of the discussion section.
