# Supplementary material for: Toward secure mobile applications through proper authentication mechanisms
Source: PLoS One. 2024 Dec 5;19(12):e0315201. doi: 10.1371/journal.pone.0315201 (PMC11620593; doi:10.1371/journal.pone.0315201)
Supplement: Attachment — Submitted filename: PLOS.docx [file pone.0315201.s003.docx]

The manuscript may be accepted in its present form.
